# The same, only different: Using physician billing data from four provincial payment systems to describe family physician practice patterns in Canada.

**DOI:** 10.23889/ijpds.v7i3.1849

**Published:** 2022-08-25

**Authors:** Ruth Lavergne, Sandra Peterson, David Rudoler, David Stock, Emily Marshall

**Affiliations:** 1 Dalhousie University; 2 University of British Columbia; 3 Ontario Shores Centre for Mental Health Sciences

## Objectives

In Canada physician payment systems and associated billing data vary across provinces. We used linked billing data to develop comparable measures of family physician (FP) service volume, continuity, and comprehensiveness in each of four provinces, with the goal of describing changing patterns over time, relevant to workforce planning policy.

## Approach

We accessed linked population and physician registry data, vital statistics, physician billing data, hospital and emergency department records, and (where available) laboratory and prescription drug records in four Canadian provinces from 1996/7 to 2017/8. We tracked changes in primary care physician service volume (patient visits), continuity, and comprehensiveness over two decades, and explored the impacts of career stage and graduation cohort on patterns observed. We also quantified changes in the volume of services that required FP coordination, review, administration, and/or follow-up, reporting changes in service volume per-capita, per community-based family physician and per physician visit.

## Results

Visit volume and continuity per provider fell over time in the four provinces examined. Visits increased with years in practice until mid-to-late-career and declined into end-of-career. We found no relationship between graduation cohort and practice volume, continuity, or comprehensiveness of care. Over this time period, the number of FPs per-capita has grown, but the number in comprehensive, community-based practice has remained constant. While primary care visits have declined, the number of prescriptions, lab tests, emergency department visits, and specialist visits per capita have all increased. When expressed per community-based FP, and particularly per community-based FP patient visit, the increases in workload range from 36.1% for lab tests to 55.0% for ED follow-up. Increases in service volume are greatest among patients ages 80 and older, a rapidly-growing population segment.

## Conclusion

Linked data capturing changes in practice patterns and workload suggest that even with an increasing per-capita supply of family physicians, additional resources will be needed to ensure all patients can access comprehensive primary care. Information on the various roles family physicians fill and demographic change will strengthen workforce planning.

